# Metabolic Insight of Neutrophils in Health and Disease

**DOI:** 10.3389/fimmu.2019.02099

**Published:** 2019-09-20

**Authors:** Sachin Kumar, Madhu Dikshit

**Affiliations:** ^1^Pharmacology Division, CSIR-Central Drug Research Institute, Lucknow, India; ^2^Translational Health Science and Technology Institute, Faridabad, India

**Keywords:** neutrophil functions, glycolysis, TCA cycle, pentose phosphate pathway, glutaminolysis, fatty acid oxidation, metabolic adaptation, diseases

## Abstract

Neutrophils are the most abundant, short lived, and terminally differentiated leukocytes with distinct tiers of arsenals to counter pathogens. Neutrophils were traditionally considered transcriptionally inactive cells, but recent researches in the field led to a paradigm shift in neutrophil biology and revealed subpopulation heterogeneity, and functions pivotal to immunity and inflammation. Furthermore, recent unfolding of metabolic plasticity in neutrophils has challenged the long-standing concept of their sole dependence on glycolytic pathway. Metabolic adaptations and distinct regulations have been identified which are critical for neutrophil differentiation and functions. The metabolic reprogramming of neutrophils by inflammatory mediators or during pathologies such as sepsis, diabetes, glucose-6-phosphate dehydrogenase deficiency, glycogen storage diseases (GSDs), systemic lupus erythematosus (SLE), rheumatoid arthritis, and cancer are now being explored. In this review, we discuss recent developments in understanding of the metabolic regulation, that may provide clues for better management and newer therapeutic opportunities for neutrophil centric immuno-deficiencies and inflammatory disorders.

## Introduction

Neutrophils, the most abundant leukocytes in human, are commonly considered as terminally differentiated effector cells (1–3). These cells reach early at the site of insult to diminish intruders and further direct the adaptive immune responses (2, 4). Indeed neutropenic patients exhibit higher susceptibility to recurrent infections (2, 3). While, aberrant neutrophil responses also lead to tissue damage and are associated with pathological conditions like sepsis, asthma, ischemia-reperfusion injury, and rheumatoid arthritis (2, 3). Neutrophils are produced in the bone marrow through a tightly regulated process of granulopoiesis. Neutrophils perform diverse functions including phagocytosis, oxidative burst, neutrophil extracellular traps (NETs) to execute microbial killing (2, 5, 6). These functions often rely on cytoskeleton reorganization and energy, but for-long neutrophils were believed to depend mainly on glycolytic metabolism (7, 8). Successively, most of the neutrophil functions were studied only in the presence of glucose that delayed identification of other metabolic pathways in neutrophil biology.

Interestingly in the last decade, a mere dumb suicidal killer view about these cells was challenged due to the identification of a number of novel functions and their crosstalk with other immune cells (1–3, 9). Most vibrant and prominent change in the field of neutrophil biology has been the identification of distinct subset heterogeneity and cellular plasticity (10–12), that for a long time remained ambiguous possibly because of their short-lived nature and high turnover. Furthermore, though initially considered as transcriptionally inactive, recent research in the field has also suggested transcriptional regulations even in the mature neutrophils during their transit to blood, under inflammatory environment, activation, and NETosis (13–15). Furthermore, new findings have strengthened the concept that neutrophils are critical regulator of immunity.

**Figure 1:**
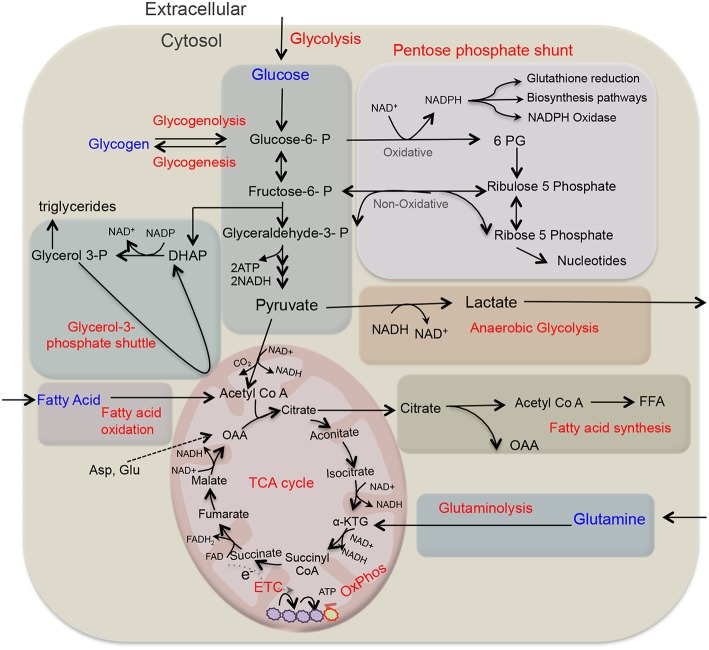
Overview of key metabolic pathways in neutrophils. Glycolysis is a major metabolic pathway in neutrophil cytosol, which converts glucose to pyruvate through a series of enzymes and reaction. In the absence of oxygen through anaerobic glycolysis, pyruvate gets reduce to lactate, and secreted out. While in the presence of oxygen, pyruvate participates in TCA cycle by its conversion to acetyl-coenzyme A (acetyl Co A) that provides reducing energy intermediates NADH and FADH2 which trough ETC generate ATPs. Neutrophils also utilize additional pentose-phosphate pathway (PPP) by using glucose-6-phosphate, an intermediate of the glycolytic pathway, as an entry point and using oxidative and non-oxidative phases produce NADPH and riboses that subsequently generate nucleotides. NADPH is critical as key NADPH oxidase dependent ROS generation in neutrophils and also regulates redox signaling. Depending on glucose abundance, glycogen stores get enriched in neutrophils that on-demand provides glucose based glycolytic intermediate supply. TCA cycle intermediate citrate through fatty acid synthesis (FAS) can lead to the generation of free fatty acid, while these endogenous FFA or transported from the extracellular environment through FAO pathway yields acetyl-CoA that fuels TCA cycle and produce significantly more energy in form of ATPs. Glutamine through glutaminolysis produces α-ketoglutarate and thus supports the TCA cycle. In addition, neutrophils also utilize glycerol-3- phosphate shuttle to generate NAD+ from NADH and helps in maintaining mitochondrial membrane potential. ATP, adenosine triphosphate; ETC, electron transport chain; FAO, fatty acid oxidation; NADPH, the reduced form of nicotinamide adenine dinucleotide phosphate; ROS, reactive oxygen species.

Cellular metabolism plays a decisive role in the function and plasticity of diverse immune cells including T cells and macrophages (16, 17), though metabolic regulations in neutrophil biology are continuously unfolding. Recent studies seem to challenge the commitment of neutrophils only to glycolysis, as different metabolic routes (Figure 1) including tricarboxylic acid (TCA) cycle (also known as Krebs cycle), oxidative phosphorylation (OXPHOS), pentose phosphate pathway (PPP), fatty acid oxidation (FAO) are being recognized to fulfill the energetic, biosynthetic, and functional requirements of neutrophils (18–23). Importantly, these cells are exposed to a variety of metabolic fuels in different organs and also at the site of infection/inflammation. Neutrophils use carbohydrates, proteins, lipids, as well as amino-acids for energy productions and uptake them by diverse transporters, and also respond to nutrients by binding to the receptors (24). Neutrophils in respond to diverse stimuli by enhancing the uptake of glucose, suggesting functional role of glucose metabolism in modulating neutrophil functions. Neutrophils exhibit differences in the expression of GLUT1, GLUT3, and GLUT4 under resting and stimulated conditions (25). Neutrophils also express cholesterol and lipid receptors including LDL receptor (LDL-R), scavenger receptor and cholesterol efflux transporter like ATP-binding cassette transporters ABCA1 and ABCG1, that regulate neutrophil adhesion and activation (26, 27). Recent studies have identified role of free fatty acid signaling in neutrophil activation and functions. Various fatty acid receptors including free fatty acid receptor-1 (FFAR1/GPR40), free fatty acid receptor 2 (FFAR2/GPR43), and GPR84 that function against LCFA (>C12), SCFA (C2-C6), and MCFA (C7-C12) are expressed on neutrophils (28). Neutrophils also express Toll-like receptors to detect pathogenic stimuli and also lipoproteins (29). In addition, role of glutamine and arginine are investigated for their effect on neutrophil functions, while others have not received much attention. Still, information on these surface receptors and transporters in neutrophil metabolic adaptations remain limited. Hopefully technical advancements in the field in relation to metabolomics will pave way for the better understanding of neutrophil production, differentiation and pivotal functions viz a viz neutrophil metabolism. Recent developments in the field suggest that neutrophils adapt to different metabolic pathways in the presence of inflammatory signals or under diverse disease conditions (18, 21). This review focuses on key neutrophil functions and their dependence on different metabolic pathways. We further discuss that how does metabolic reprogramming impact neutrophil biology under different environments or disease conditions.

## Major Metabolic Pathways in the Neutrophils

Recent studies identified that neutrophils by utilizing various metabolic intermediates, formed by different metabolic pathways operative in the cytosol and mitochondria, meet their energy requirements (18, 20, 30–33). Though these metabolic pathways have been extensively reviewed earlier (16, 17), here we briefly summarized key metabolic routes important during neutrophil differentiation and functions (Table 1). During glycolysis, glucose is enzymatically converted to pyruvate in the cytosol, providing relatively low level of ATP and NADH. Under anaerobic condition, lactate is formed from pyruvate, and this conversion is catalyzed by the enzyme lactate dehydrogenase. While in the presence of oxygen, pyruvate forms acetyl coenzyme A, which enters TCA cycle in the mitochondria to efficiently generate energy (ATP) through electron transport chain (ETC) (16, 17). The PPP or hexose monophosphate (HMP) shunt utilizes glycolytic intermediate glucose-6-phosphate to generate ribose- 5-phosphate and NADPH in the cytosol. NADPH is a key substrate for NADPH oxidase (NOX2) to sustain ROS production by the activated neutrophils (40). Under fasting conditions or due to limited availability of glucose, cells adapt to fatty acid metabolism. Mitochondrial fatty acid β-oxidation (FAO) converts fatty acids to acyl-CoAs to yield acetyl-CoA that enters the TCA cycle. Glutamine is yet another metabolic substrate utilized by neutrophils particularly under glucose-limiting conditions (41–43). Glutaminolysis via glutamate dehydrogenase forms α-ketoglutarate that fuels TCA cycle (41). Metabolic plasticity during diverse stress thus helps to meet the energy requirement by efficiently diverting the intermediate metabolites being generated by the operative metabolic pathways (44).

**Table 1:** Key metabolic pathways regulating distinct neutrophil functions.

**Neutrophil function**	**Metabolic pathways**	**Brief summary**	**Reference**
Differentiation 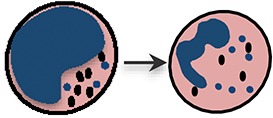	FAO, TCA, OxPHOS	During differentiation, Glycolysis & autophagy decline. Modulation of FAO, TCA, and mitochondrial respiration regulate neutrophil differentiation.	(18, 19, 22, 34, 35)
Energetics 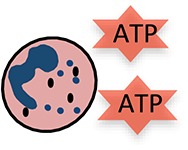	Glycolysis	Neutrophil ATP level completely abolishes by glycolysis inhibitors, While remains insensitive to mitochondrial respiratory chain inhibitors	(7, 8, 30, 36)
Phagocytosis 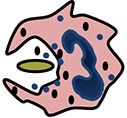	Glycolysis	Glycolytic inhibitors completely diminish neutrophil phagocytic functions.	(7, 8)
ROS/RNS 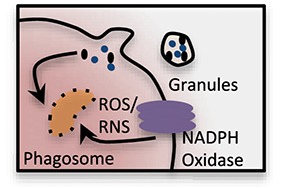	PPP, ETC (complex I, III)	PPP potentiates ROS by providing NADPH, a substrate of NADPH Oxidase, while complex I and III inhibition cause generation of mitochondrial ROS.	(21, 30, 37, 38)
NETs 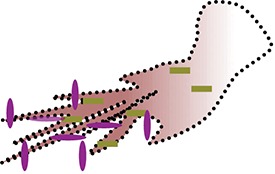	Glycolysis and PPP	Glucose uptake increases during NETosis and Glycolysis (2-DG) and PPP (6-AN) inhibitors mitigate NETs formation	(20, 21)
Chemotaxis 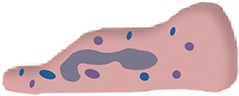	Mitochondria, Purinergic signaling	ATP and purinergic signaling at front governs neutrophils chemotaxis. Mitochondria with high Δψm localize at front & promote purinergic signaling. FCCP blocks chemotaxis.	(30, 39)
Apoptosis 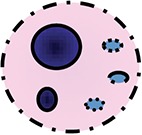	Mitochondria	Mitochondria function as redox, Ca^2+^ sensor. Defective Δψm initiates release of proapoptotic factors into the cytoplasm and cause apoptosis.	(30–32)

*6-AN, 6-amino-nicotinamide; ATP, Adenosine triphosphate; 2-DG, 2-deoxy-glucose; Ca^2+^, Calcium; ETC, Electron transport chain; FAO, Fatty acid oxidation; FCCP, carbonil cyanide p-triflouromethoxyphenylhydrazone; Δψm, Mitochondrial membrane potential; OxPHOS, Oxidative phosphorylation; PPP, Pentose phosphate pathway; ROS, Reactive oxygen species; TCA, tricarboxylic acid cycle*.

Furthermore, the role of mitochondria has been remained less understood in neutrophils and is discussed here. Initial investigations using electron microcopy have observed very few functional mitochondria in mature neutrophils (45, 46). Later other studies have implied the dispensable role of mitochondria for respiration, or energy production, as mitochondrial respiration inhibitors failed to alter ATP levels in neutrophils (8, 32, 36). As neutrophils utilize oxygen mainly for NOX-2 dependent superoxide generation, functional investigations on mitochondrial involvement have remained challenging. Recent advancements in mitochondrial probes, Seahorse assay for oxygen consumption rate (OCR) and extracellular acidification rate (ECAR) analysis and metabolomics methods are identifying novel role of mitochondria in neutrophil biology. Consistently, neutrophils contain significantly low levels of oxidative phosphorylation complexes (47) and mitochondrial enzymes including glutamate dehydrogenase (GDH) and fumarase (32). In contrast, Fossati et al. have challenged the view of so-called few functional mitochondria in neutrophils, by using MitoTracker Red/JC-1 and demonstrated presence of a complex mitochondrial network in the neutrophils (30). This study has revived the thrust of research on mitochondria and identified specific and differential role of mitochondria in respiratory burst, chemotaxis, and apoptosis (30). Various studies have also demonstrated role of purinergic signaling (derived from purine nucleotide/nucleoside analogs such as adenosine and ATP) in the activation, migration, and other functions of neutrophils (48–50). Together, functional role of mitochondria as the redox regulator, rheostat for survival and migration have been identified in neutrophils (30). These functions and their metabolic regulation are being further discussed in the following sections.

## Metabolic Regulation of Neutrophil Functions

Neutrophils, the highly dynamic cells, perform different functions in diverse microenvironments. Though the glycolytic view prevailed very long for neutrophils, recent researches have however, identified unique metabolic decisions of neutrophils during differentiation, survival, and apoptosis. Multiple approaches using diverse substrates, inhibitors, and/or knockout of genes affecting metabolism have identified utility of different metabolic pathways during neutrophil development and various functions (18–23). Key proteins and genes that are involved in the metabolic regulation during neutrophil development and performance of various functions are listed in Table 2. Specific modulations of TCA cycle, ETC, PPP, glutaminolysis, and fatty acid metabolic pathways seem to regulate neutrophils under patho-physiological settings. Following sections describe major metabolic requirements of neutrophils during development, functions, and survival.

**Table 2:** Summary of distinct genes/proteins involved in metabolism and regulating neutrophil functions.

**Gene/Protein**	**Metabolic function**	**Approaches/models utilized**	**Neutrophil Phenotype/comments**	**References**
AK2	Regulates the energy metabolism in mitochondria through homeostasis of adenine nucleotides	shRNA induced knock-down of adenylate kinase 2	Blocked granulocyte differentiation, elevated glucose consumption but abrogated mitochondrial metabolism	(19)
NAMPT	Essential enzyme for NAD+ biosynthesis, maintain TCA and FAO pathways	NAMPT and its substrate vitamin B3 (nicotinamide) treatment	Induced granulocytic differentiation of CD34+ and HL-60 cells	(34)
Atg5	Involved in autophagosome formation, down products provide inputs to cellular metabolism and energetics	Atg5fl/fl-Lyz2Cre/Cre mice, ShRNA-KnockdownAtg5flox/flox-Lyz2Cre/Cre mice	Increased proliferation rate in the neutrophil precursor cells and neutrophilia Decrease in inflammation due to defective degranulation and ROS	(35, 51)
Atg7	Essential for Autophagy, provides free fatty acids	Vav-cre 3 Atg7f/f, *Cebpa* promotor *cre*	Reduced neutrophil differentiation	(18)
FAS	Involved in lipogenesis	*FASlox/lox-Rosa26-CreER* mice	Dramatic loss of neutrophils due to apoptosis	(52)
PexRAP	Terminal step enzyme in ether lipid synthesis	Inducible Knockout using *Rosa26-CreER* promoter	Leukopenia with loss of neutrophils	(52)
GPR43/ FFAR2	Receptor for short-chain fatty acids	Constitutive free fatty acid receptor 2 deficient mice	Reduced neutrophil recruitment, high susceptibility to inflammation	(53, 54)
G6PD	Regulate switch of glycolysis toward pentose phosphate pathway, causes NADPH dependent depletion of glutathione	G6PD deficient patientsG6PD-deficient mutant (G6PD^mut^) mice	Defects in ROS generation and NETosis with induced susceptibility to infection Hematopoietic G6PD^mut^ exhibited improved glucose tolerance, insulin sensitivity with low FFAs	(55) (56)
G6pc3	Glucose-6-phosphatase- β that hydrolyzes glucose-6-phosphate	Patient with G6Pase-β deficiency *G6pc3* deficient mice	Severe congenital neutropenia Enhanced apoptosis, neutropenia, Defective superoxide generation, chemotaxis and calcium flux	(57–60)
polg	Mitochondrial DNA polymerase regulates OXPHOS	CRISPR/Cas9 mediated neutrophil-specific knockout in Zebra fish	Reduced the velocity of neutrophil interstitial migration	(61)
SDH	Involved in TCA cycle and ETC	Patients with mutations in sdh	Reduced constitutive apoptosis	(22)
Pdh2	Regulates glycolytic flux and glycogen stores	Phd2^fl/fl^ LysM-Cre (myeloid-specific loss of Phd2)	Increased neutrophil activation, motility, functional capacity, and survival due to enhanced glycolytic capacity, intracellular ATP, and glycogen	(62)
HIF-1a	Oxygen sensing and glycolytic flux	Deletion of HIF-1α using LysM-Cre	Defective glycolysis and ATP generation. Low expression of granule proteases, decline in antimicrobial activity, and survival	(63, 64)
mTOR	Energy and nutrient sensor, regulates Glycolysis, OxPHOS, and Autophagy	ShRNA mediated knockdown of mTOR complex proteins, pharmacological inhibition	Inhibits neutrophil chemotaxis, differentiation, and NETosis	(35, 65, 66)

*AK2, adenylate kinase 2; Atg5, Autophagy related 5; Atg7, Autophagy related 7; ATP, Adenosine triphosphate; FAO, Fatty acid oxidation; FAS, Fatty acid synthase; G6Pase-β, Glucose-6-phosphatases- β; G6PD, Glucose-6-phosphate dehydrogenase; GPR43/FFAR2, G-protein coupled receptor 43; HIF-1α, Hypoxia-inducible factor-1α; mTOR, Mammalian target of rapamycin; NAD, Nicotinamide dinucleotide; NAMPT, Nicotinamide phosphor-ribosyltransferase; OxPHOS, Oxidative phosphorylation; Pdh2, Prolyl hydroxylase 2; PexRAP, Peroxisomal Reductase Activating PPAR; polg, Mitochondrial DNA polymerase gamma; PPAR, Peroxisome proliferator-activated receptor; SDH, Succinate dehydrogenase; TCA, Tricarboxylic acid cycle*.

### Development and Differentiation

Neutrophils are normally produced in the bone marrow at a very high rate of 0.5–1.0 × 10^11^ cells per day through a highly controlled process of granulopoiesis (67) that includes generation of granulocyte-monocyte progenitors (GMPs) from hematopoietic stem cells (HSCs) and multipotent progenitors (MPPs). GMPs generate mature neutrophils through distinct developmental stages like myeloblasts (MBs), promyelocytes (PMs), myelocytes (MCs), metamyelocytes (MMs), band cells (BCs) (67). Interestingly, neutrophil differentiation can be drastically increased under stress or infection conditions, that is critical to maintain patho-physiological demands. Several lines of evidence suggested distinct metabolic regulation of neutrophil development with specific role of mitochondrial respiration, TCA cycle and oxidative phosphorylation (OXPHOS) in terminally differentiated neutrophils (19, 22, 34). In particular, deficiency of adenylate kinase 2 (AK2), that regulates adenine nucleotides homeostasis, exhibited defective mitochondrial activity (19). AK2 deficiency blocked granulocyte differentiation in promyelocytic HL60 cell line that was associated with elevated glucose consumption and significant accumulation of lactate and pyruvate suggesting a metabolic shift in the granulocyte progenitors leading to differentiation block (19). In another study, nicotinamide phosphor-ribosyltransferase (NAMPT), an essential enzyme for nicotinamide dinucleotide (NAD+) biosynthesis, maintained TCA and fatty acid oxidation (FAO) metabolic pathways, and triggered G-CSF induced granulopoiesis (34). Furthermore, NAMPT substrate vitamin B3 (a metabolic precursor of NAD) also induced neutrophilic differentiation, suggesting a decisive role of the NAD+ metabolic pathway in neutrophil development (34).

The role of FAO in neutrophil differentiation has also been observed through autophagy, a key process of auto-degradation that provides free fatty acids (FFA) (18, 35, 51). Interestingly, autophagic activity was observed to consistently reduce during neutrophil differentiation (18, 35). Deficiency of Atg5 (Autophagy related 5 protein) in neutrophilic lineage exhibited increased and more rapid neutrophil production leading to neutrophilia *in vivo*, without affecting functions like phagocytosis, ROS generation, and migration (35). In contrast, Bhattacharya et al. suggested reduced degranulation, ROS production and neutrophilic inflammation (51). These conflicting findings might be due to the utilization of different experimental approaches, emphasizing for better understanding of the role of autophagy in neutrophil differentiation. In a recent study, Riffelmacher et al. utilized *Cebpa* promotor *cre to delete* autophagy gene *Atg7* at GMP-MB-stage in neutrophil precursors to evaluate early granulopoiesis and avoiding any HSC level defect. *Atg7* deletion also led to neutrophilia with an accumulation of immature neutrophils, as deficiency of Atg7 or Atg5 caused differentiation defect in the earliest committed neutrophil precursors (MBs-MCs) (18). Further investigations focusing on metabolic regulation during neutrophil development and differentiation revealed a decline in most of the genes involved in glycolytic pathway from myeloblasts (MBs) to neutrophils, while mitochondrial content up-surged 2 fold during neutrophil differentiation (18), this was surprising and in contrast to the glycolytic view of mature neutrophils. Atg7 deficient neutrophil precursors exhibited increased glycolytic activity but impaired mitochondrial respiration, decreased ATP production, and accumulated lipid droplets, again suggesting autophagy dependent metabolism switch from glycolysis to FAO and mitochondrial respiration during neutrophil development (18). Consistently, conditional ablation of Atg7 also depleted FFA like palmitic, oleic, and eicosanoic acids that induced neutrophilia due to increase in immature neutrophils with inefficient phagocytosis and bacterial killing. Moreover, inhibition of FAO using etomoxir also led to the accumulation of lipid droplets and suppression of neutrophil maturation. Similarly, inhibition of autophagy-mediated lipid degradation using lipase inhibitors DEUP/orlistat caused a glycolytic shift in the metabolism and led to defective neutrophil differentiation. Importantly, administration of fatty acids or pyruvate that fuel the mitochondrial respiration rescued differentiation of autophagy-deficient neutrophil precursors (18). These findings thus identified importance of oxidative phosphorylation/mitochondrial respiration and fatty acid metabolism during neutrophil development and maturation.

### Phagocytosis

A characteristic function of phagocytic cells, such as neutrophils and macrophages, is intricately regulated by various membrane receptors and intracellular signaling. During phagocytosis, receptor-mediated uptake of an opsonized pathogen into plasma membrane-derived vacuoles, further lead to the fusion of granules and lysosomes with phagosomal membrane to deliver anti-microbial peptides and proteases.

Early studies have suggested that neutrophil phagocytic functions predominantly depend on glycolytic pathway, based on their sensitivity to glycolysis inhibitors, sodium iodoacetate (SIA) and sodium fluoride and insensitivity to mitochondrial respiratory chain inhibitors, potassium cyanide (KCN), and antimycin A (7, 8). Furthermore, neutrophils depend on glucose for the energy requirement, as both sodium iodoacetate and 2-deoxyglucose (2-DG) diminished ATP levels. 2-DG competitively blocked the production of glucose-6-phosphate (G6P) thus both glycolysis and PPP pathways were inhibited, while SIA irreversibly inhibited glyceraldehyde-3-phosphate dehydrogenase (GAPDH) enzyme thus only glycolytic pathway was stopped. Importantly antimycin A, sodium azide, or cyanide had a negligible effect on ATP generation and oxygen consumption in phagocytizing neutrophils (7, 8), suggesting a dispensable role of mitochondrial metabolism in phagocytosis. Consistently, Fossati et al. have also observed that phagocytosis remains un-affected in the presence of FCCP or oligomycin, that disrupt mitochondrial membrane potential and Fo-ATPase, respectively (30). Interestingly, inflammatory environment that is commonly associated with low oxygen tension seems to sustain neutrophils on glycolysis (8). Phagocytosis also depends on stored glycogen in the neutrophils (7) so as to provide glucose through glycogenolysis for phagocytic functions (7, 8, 30, 68). Molecular regulations of glycogenolysis, glucose uptake, and glucose utilization in phagocytosing neutrophils warrant further investigations for better understanding.

### Oxidative Burst

Respiratory burst or superoxide (${\text{O}}_{2}^{-}$) generation by the neutrophils is catalyzed by NOX-2, a multi-subunit enzymatic complex by utilizing oxygen and NADPH (5, 69, 70). Importantly, patients with the chronic granulomatous disease (CGD) that exhibit functional mutations in NOX2 subunit, counter life-threatening infections (70). NOX-2 activation involves translocation of cytoplasmic subunits including p47^phox^ to the membrane. Rac 2, a protein activated only in GTP bound state initiates the NOX-2 activation and is an energy dependent process. NADPH, the NOX-2 substrate is produced by PPP utilizing G-6-P, an intermediate generated by glycolysis or glutaminolysis. A few studies have implied the additional role of mitochondria in ROS production, as complex I inhibition with rotenone or metformin and mitigation of complex III with myxothiazol or antimycin A, lead to an increase in superoxide and hydrogen peroxide generation (37, 38).

Neutrophils primarily depend on a rapid consumption of oxygen by NOX2 system for superoxide and ROS generation, thus ROS production can be intriguingly mimicked by OCR in neutrophils (71). Diphenyleneiodonium (DPI), a NOX-2 inhibitor and 2DG, inhibitor of glycolysis completely suppress PMA dependent increase in OCR (71), thus confirming role of glycolysis in NOX-2 activation/ROS generation. Rice et al. recently dissected involvement of diverse metabolic pathways in ROS generation (72). 2DG mediated block of glycolysis only reduced the initial-earlier phase OCR, in contrast the later phase of OCR profile remained unaffected. Interestingly, mitochondrial inhibition using rotenone/antimycin A, FCCP, and fatty acid metabolism inhibition by etomoxir, led to dramatic reductions in OCR during 2DG treatment (72). This suggests requirement of glucose metabolism for the early phase of high and intense ROS production, while both fatty acid metabolism and mitochondrial function facilitated prolonged H_2_O_2_ production during the late phase.

Neutrophils from glycogen storage disease (GSDs) patients having glucose-6-phosphate transporter (*G6PT*) deficiency also exhibited impaired ROS generation, suggesting the importance of glucose homeostasis in NOX-2 activation (57). Interestingly, neutrophils meet their requirement of energy by utilizing different metabolic intermediates/nutrients during phagocytosis and chemotaxis (68, 73). Various soluble chemotaxins accelerate the transmembrane glucose uptake without affecting stored endogenous glycogen (73). In a recent study, glycolytic enzyme 6-phosphofructo-2-kinase (PFK-2) was found to localize with NOX-2 following neutrophil activation. While inhibition/depletion of PFK-2 reduced the rate of glycolysis and also NOX2 activity (74). Surprisingly, NOX2 inhibition also reduced the rate of PFK-2 catalyzed glycolysis, this revealed an unexpected function of NOX2 in the activation dependent increase in glycolytic metabolism through PFK-2 (74). Interestingly, addition of α-ketoglutarate to the neutrophil suspension significantly increased superoxide and H_2_O_2_ generation as well as intracellular pyruvate, asparagine, glutamine, aspartate, and glutamate (75). Similar results were also observed following treatment with pyruvate on ROS production (76), suggesting metabolic adaptation/plasticity of neutrophils to utilize distinct metabolic intermediates for catabolic and anabolic processes. Moreover, role of short chain fatty acids (SCFA), in particular acetate via GPR43 receptor has also been suggested in enhanced ROS generation and phagocytosis (77).

### Neutrophil Extracellular Traps (NETs)

Neutrophils release chromatin traps along with antimicrobial granular contents through NETosis to kill and prevent the dissemination of pathogens (6). NETosis is also energy driven catabolic process that requires inter-mixing of cytoplasmic granules with decondensed nuclear chromatin, and expulsion of filamentous traps (6, 21). Mediators promoting NETosis have been categorized on the basis of their dependence/independence on ROS. Recent studies are focusing on finding the metabolic requirement of NETosis (20, 21), which suggest its dependent on glucose but not on glutamine (20). Consistently uptake of glucose was increased during phorbol myristate acetate (PMA) induced NETosis (20). Moreover, ROS, a known driver of NETosis was less dependent on glucose during the initial phase of chromatin decondensation (20). Rodriguez-Espinosa et al. thus proposed an essential role of glucose and glycolysis for the second phase of NETosis i.e., NETs release (20). Indeed, 2-DG that diminished NETosis (20), simultaneously inhibited glycolysis as well as PPP. Subsequent study conducted by Azevedo et al. revealed a metabolic shift toward PPP during NETosis (21), which was coupled with increased activity of glucose-6-phosphate dehydrogenase (G6PD) that diverts glycolytic intermediate G6P to PPP. Consistently, G6PD inhibitor, 6-aminonicotinamide (6-AN) has also reduced ROS production and DNA release in PMA and amyloid fibrils stimulated cells, suggesting G6P as a fuel for NOX2 activation (21). Though incomplete inhibition of ROS as well as NETs with G6PD inhibitor advocates the possibility of additional NADPH source than PPP or concurrently active glycolytic pathway, warranting further investigations. It is also important to mention that due to short life of neutrophils most of metabolic studies are conducted using pharmacological agents, which also have non-specific effects.

Until recently, it has been well-accepted that under anaerobic conditions glucose produces lactate as metabolic waste. Recently, two independent breakthrough studies using ^13^C-lactate tracing identified that lactate can be converted to pyruvate and fuel metabolism through TCA cycle (78, 79). Lactate can be converted back to glucose in the liver through Cori cycle (78, 79). Neutrophils are commonly present in hypoxic and inflammatory sites that are often enriched with lactate, but role of lactate in neutrophil function is relatively less explored. Rodriguez-Espinosa et al. have linked PMA induced lactate release and NETs formation (20). In light of the recent paradigm shift in role of lactate in metabolism, lactate might play a unique role in NETosis. Consist to this, Alarcon et al. demonstrated that d(–) lactic acid, which can be formed by bacteria, induced NETosis in bovine neutrophils (80). Significant changes in the lactate level during diverse metabolic and inflammatory diseases, like diabetes, sepsis etc, might modulate neutrophil responses and NETosis. Recent study conducted in our lab has demonstrated that lactate was formed during PMA and A23187 induced NETosis, and oxamate, a LDH inhibitor reduced NETs release by these mediators (81). Moreover, previous study from our lab has also demonstrated that oxidized LDL though TLR pathway induced NETosis in human PMNs (82), suggesting a possible role of FAO in NETosis and neutrophil-mediated tissue damage. NETosis has been linked with both physiological and pathological conditions (3), thus necessitating a proper understanding of the metabolic requirements for improved understanding and for developing future therapeutic interventions.

### Chemotaxis

Participation of neutrophils in immunity is highly dependent on chemotaxis, a process of cell migration toward intruder/injury site (83). Importantly, chemotaxis is a coordination of cytoskeleton reorganizing events including adhesion, polarization following actin-enriched pseudopod formation in the front and myosin-dependent contraction toward uropod (83, 84). Together these cytoskeleton reorganization events are energy dependent and well-supported by small GTPases during neutrophil migration (84, 85).

Though in neutrophils primarily dispensable for bioenergetics, mitochondria and TCA cycle enzymes has been observed to regulate chemotaxis (33, 39, 61). FCCP mediated significant inhibition of neutrophil migration suggested the role of mitochondrial signaling in chemotaxis of neutrophils (30). Further study revealed that neutrophils release ATP at the front that amplifies purinergic signaling through P2Y2 nucleotide receptors (39). Indeed, in response to chemotactic cues mitochondria sense and release ATP to further stimulate P2Y2 receptors and promote mTOR signaling to fuel the mitochondrial activity at the leading edge (33). Activated mitochondria displaying high calcium uptake and Δψm localize to the front and deliver the ATP to promote purinergic signaling (33). Furthermore, inhibition of mitochondrial ATP production by cyanide, rotenone, and CCCP, an uncoupler completely blocked chemotaxis by mitigating gradient sensing and speed through alternative purinergic signaling (33). A recent study identified direct evidence of mitochondrial role in neutrophil migration, as neutrophil-specific disruption of mitochondrial DNA polymerase, *polg* led to reduced motility of neutrophils in Zebra fish (61). In yet another study, prolonged treatment with oligomycin, inhibited chemotaxis (30), suggesting an essential role of Fo-ATPase to uphold chemotaxis.

Iso-citrate dehydrogenase (IDH) is an important enzyme in cellular metabolism that decarboxylates isocitrate to α-ketoglutarate in TCA cycle. IDH-1 mutant neutrophils exhibited impaired chemotaxis, suggesting the importance of TCA metabolism in neutrophil function (86). Short-chain fatty acids (SCFAs) including acetate, propionate, and butyrate via L-selectin stimulation induced neutrophil migration *in vivo* (87). SCFAs by engaging with G-protein coupled receptor 43 (GPR43, also known as FFAR2) induced neutrophil chemotaxis (53), additionally GPR43 deficient neutrophils failed to migrate to propionate or GPR43 agonist phenylacetamide-1 (53, 54). SCFAs including acetate catabolize to acetyl-Co-A that generate a higher amount of ATP molecules through TCA and OXPHOS. Thus, these studies reveal role of mitochondria and TCA cycle in neutrophil chemotaxis. Moreover, neutrophils migrate to all the organs/tissues in the body, it is well-known that organs exhibit distinct preference for the metabolic fuels. How does diverse intermediates of distinct metabolic pathways in various organs impact neutrophil populations and their functions remain to be defined.

### Apoptosis

Neutrophil homeostasis is governed by the clearance of exhausted cells through the apoptotic process, while uncontrolled apoptosis can lead to neutropenia. Apoptosis is an energy-dependent process (88), similarly prolonged survival of neutrophils might be a result of the change in energy homeostasis and metabolism (89). Intriguingly, G-CSF exerts an anti-apoptotic effect through mitochondria-dependent mechanisms (31). In spite of minimal involvement in energetics, mitochondria in neutrophils maintain the membrane potential (Δψm), which depends on proton gradient derived from respiratory chain complexes and OXPHOS (47). Complex III can receive electrons from glycolysis via the glycerol-3-phosphate shuttle that helps to maintain Δψm using mitochondrial glycerol phosphate dehydrogenase (mGPD) enzyme in neutrophils (47). While a loss of Δψm in neutrophils precedes the phosphatidylserine exposure and initiates the release of cytochrome C and other pro-apoptotic factors into the cytoplasm to prime apoptosis (30–32).

A study using patients with mutations in succinate dehydrogenase (SDH) encoding gene indicates its role in neutrophil apoptosis (22). SDH oxidizes succinate to fumarate in the TCA cycle and also functions as ubiquinone oxidoreductase in ETC complex II (22). Neutrophils from these patients exhibited higher succinate levels, and reduced constitutive as well as hypoxia induced apoptosis, in an HIF-1α independent manner (22). Consistently, treatment with 3-nitropropionic acid, an irreversible SDH inhibitor led to reduced constitutive apoptosis in neutrophils from healthy individuals (22), implying role of TCA cycle intermediates in neutrophil apoptosis.

Lodhi et al. identified an important role of fatty acid synthase (FAS)-dependent *de novo* lipogenesis in neutrophil apoptosis (52). FAS catalyzes palmitate formation from malonyl-CoA. Conditional global FAS knockout mice die from neutropenic sepsis due to disrupted membrane phospholipid composition in FAS KO neutrophils. Loss of FAS impaired lipogenesis but enhanced programmed cell death without affecting granulocytic differentiation. Furthermore, peroxisomal reductase activating PPAR (PexRAP) knockout mice also exhibited loss of neutrophils, without drastically affecting other hematopoietic cells (52). Aoyama et al. have shown induction of caspase-dependent neutrophil apoptosis in the presence of SCFAs propionate and butyrate (90). In contrast, acetate induced neutrophil apoptosis was GPR43 dependent (77). Together these studies demonstrate role of FA metabolism in neutrophil apoptosis.

Enhanced neutrophil apoptosis during sepsis is associated with a decrease in glutamine concentration (91). Expectedly, glutamine supplementation protected mice from sepsis and organ failure (92–94), possibly by reducing neutrophil apoptosis (95–98). In yet another study, L-alanyl-L-glutamine led to a significant increase in alpha-ketoglutarate, pyruvate and ROS generation in neutrophils (99), while 6-diazo-5-oxo-L-norleucine (DON), a glutamine-analog that inhibits glutamine utilization reversed L-alanyl-L-glutamine effects (100). These studies, however overlooked ligand/stimuli induced functional responses. Thus, glutamine and its metabolic intermediates support functions of mitochondria and survival under diverse conditions in the neutrophils. Together these findings imply the importance of mitochondrial pathways, TCA cycle and fatty acid metabolism in neutrophil development, functions and survival.

## Major Regulators of Neutrophil Metabolism

**Figure 2:**
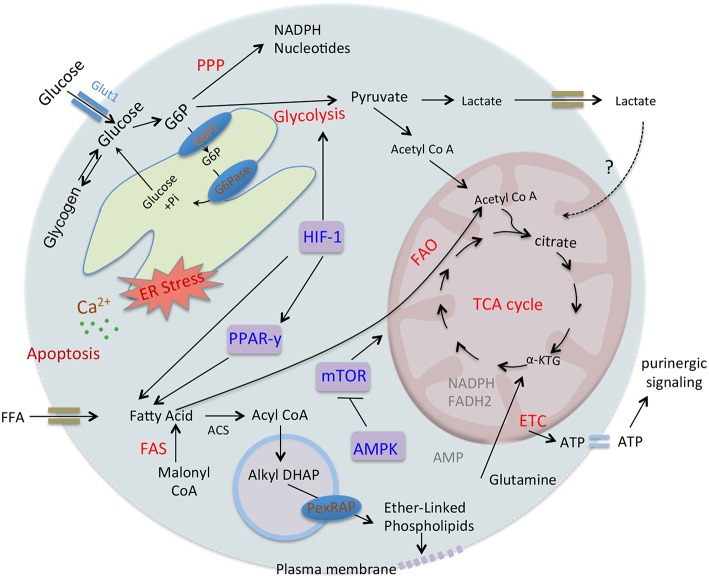
Regulation of neutrophil metabolism. Neutrophil utilizes distinct metabolic pathways including glucose oxidation, glycogen breakdown and β-oxidation of fatty acids. After transport of glucose in the cytosol, hexokinase converts it to G6P that participate in different pathways like glycolysis, PPP, glycogen synthesis, or ER cycling. Glycolysis converts glucose to pyruvate that can further utilize in TCA cycle or forms lactate. G6PT and G6Pase regulate recycling of G6P in ER and disruption of glucose/G6P balance and ER stress due to G6PT or G6Pase-β deficiencies result in impaired energy homeostasis, functions, and survivals of neutrophils. While FFA gets transport to the cytosol and autophagy also release FFA that undergoes β-oxidation and provides a high level of ATPs through mitochondrial OxPHOS pathway and regulates neutrophil differentiation. On the other hand, in neutrophils FAS and PexRAP produce peroxisomal lipids that get incorporated into the plasma membrane and thus maintaining membrane integrity and viability. Mitochondrial OxPHOS provides ATPs that also drives purinergic signaling in neutrophils. Transcriptional regulator HIF-1α regulates glucose transport as well as enzymes involved in the glycolysis. HIF-1α regulates PPARy, that also increases glucose uptake and regulates fatty acid metabolism. Interestingly, over-activation of PPARy in diverse diseases leads to defective energetics and functions of neutrophils. mTOR functions as a master regulator of mitochondrial metabolism and also regulates purinergic signaling. AMPK that senses AMP-ATP levels also controls neutrophil metabolism and functions.

Neutrophils constantly infiltrate almost all the organs and are therefore exposed to a variety of microenvironments in diverse organs/tissues. Furthermore, inflammatory sites are often associated with a low level of oxygen and glucose (101). Recent research identified key regulators of metabolic adaptability of neutrophils under these different conditions and is being discussed here (Figure 2). Hypoxia-inducible factor-1α (HIF-1α) and the mammalian target of rapamycin (mTOR) have been recognized as major regulators of metabolism in myeloid cells (63, 102). Importantly, neutrophils possess HIF-1α and factor inhibiting HIF (FIH) hydroxylase to sense hypoxia (101). Interestingly, in hypoxic environment neutrophil apoptosis is prevented by NF-κB activation (101). On the contrary, based on elevated oxygen consumption during respiratory burst, activated neutrophils at the sites of inflammation are advocated to induce oxygen depletion to promote inflammatory hypoxia (103). HIF-1α-null neutrophils exhibited low expression of granule proteases, including elastase and cathepsin G with reduced anti-microbial activity (64). In contrast, vHL-null cells with stabilized HIF-1α were observed to possess a high amount of proteases and bactericidal capacity (64). While, neutrophil development has remained unaffected by specific deletion of HIF-1α in the myeloid progenitors (63). however HIF-1α null macrophages have shown defects in hypoxia-induced Glut-1 expression (63), suggesting toward the role of HIF-1α in glucose utilization. In addition, neutrophils also express HIF-2α having overlapping functions with HIF-1α, and its expression has been up-regulated during inflammatory conditions. The specific role of HIF-2α in neutrophil apoptosis was observed using gain-of-function mutations that exhibited diminished neutrophil apoptosis (104). In contrast, HIF-2α-deficient neutrophils displayed enhanced apoptosis, leading to a reduction in neutrophil-mediated inflammation (104). HIFs are under tight regulation of oxygen tension and prolyl hydroxylases (PHDs) that cause ubiquitylation and proteasomal degradation of HIFs. Sadiku et al. demonstrated that the myeloid-specific deletion of PHD2 led to lung injury following enhanced neutrophil survival (62). Mechanistically, PHD2-deficient neutrophils displayed enhanced glycolytic capacity, generation of PPP intermediates, glycogen stores, and ATP levels. Further, glycolysis inhibition has protected these mice against enhanced neutrophilic responses, suggesting a direct connection of metabolic modulation during infection as well as inflammation (62). A recent study further confirmed that during anoxic condition, glucose and dimethyloxalylglycine (DMOG) maintained neutrophil viability and functionality for a longer period (89). Furthermore, ferroprotein sensing has been suggested to play a role in hypoxia-mediated inhibition of neutrophil apoptosis (105). Iron-chelating agents, desferrioxamine (DFO) and hydroxypyridines (CP-94) inhibited apoptosis under normoxic condition, while no effect was observed under hypoxia (105). Lactoferrin, an iron-binding protein released from activated neutrophils also enhanced neutrophil survival in the synovial fluid of rheumatoid arthritis patients (106).

mTOR, a known metabolic regulator (102), modulated chemotaxis, via mTORC2 mediated pseudopod protrusion, and cAMP accumulation at the neutrophil front (65). Furthermore, mTOR inhibition by rapamycin or PP242 reduced neutrophil chemotaxis via inhibition of chemokine-mediated ATP release. While at the uropod, A2a receptors mitigated mTORC2 signaling and blocked mitochondrial activation causing uropod retraction (33). Furthermore, rapamycin, an inducer of autophagy, mediated inhibition of mTORC1 also delayed neutrophil differentiation process (35). Consistently, pharmacological inhibition of mTOR or HIF-1α blocked NETs-dependent bacterial killing (66). Furthermore, expression of nuclear receptor peroxisome proliferator-activated receptors (PPARs) that drive fatty acid signaling, is enhanced in the neutrophils after activation (107). Moreover, sepsis patients exhibited high PPAR-y activity and reduced neutrophil chemotactic activity, which was prevented by PPAR-y antagonist (107). In addition, AMP-activated protein kinase (AMPK), a cellular energy sensor, maintains energy homeostasis via stimulation of glucose uptake and FAO in the cells. Interestingly, AMPK activation induced neutrophil chemotaxis, phagocytosis, and bactericidal activity (108). Together these studies demonstrate important metabolic regulators of neutrophil responses.

## Metabolic Adaptations in Neutrophils During Pathological Conditions

Neutrophil survival is enhanced during inflammatory conditions by modulating their apoptotic program possibly through metabolic reprogramming due to hypoxia and the nutrients availability at the inflammatory sites (101, 109, 110). Further, during distinct chronic, inflammatory and metabolic diseases, neutrophils also exhibit altered functions that are being discussed in the following section and are summarized in Table 3.

**Table 3:** Metabolic alteration responsible for neutrophil dysfunctions in disease conditions.

**Disease**	**Metabolic alteration**	**Neutrophil Phenotype/ functions**	**Molecular targeting**	**References**
Sepsis	Altered cellular bioenergetics mitochondrial and FAO pathways and atypical metabolic milieu	Paralysis of neutrophil migration and functions	Targeting PPAR-γ and purinergic signaling reversed neutrophil chemotaxis defects	(107, 111–113)
Diabetes	Diminished G6PD and glutaminase activities, high FFA and triacylglycerols, depolarized mitochondrial potential, and low ATP due to defective autophagy	Adhesion, chemotaxis, phagocytic, ROS production, and microbicidal defects	Insulin and metformin protected some of neutrophil functions	(114–116)
*Cystic fibrosis*	Excessive free glucose and amino acids, elevated mTOR signaling	Impaired degranulation and phagocytosis functions	Increase mTOR, Glut1 and PiT1, functional reprogramming with decreased intracellular glutathione	(109, 117, 118)
*Glycogen storage disease GSD-Ib*	G6PT deficiency, Disturbed glucose homeostasis with defective glucose uptake and reduced levels of G6P, lactate, ATP and NADPH	Neutropenia and neutrophil Neutropenia and neutrophil, respiratory burst	Unregulated HIF-1α and activated PPAR-γ pathway, PPARγ antagonist rescued neutrophil dysfunctions	(57)
G6Pase-β deficiency	Impaired energy homeostasis with low glucose uptake and G6P levels, enhanced ER/ mitochondrial stress	Severe congenital neutropenia due to increased apoptosis, Defective superoxide, chemotaxis, calcium flux	G-CSF rescues neutrophil defects by inducing glucose uptake and energetics	(60)
*Systemic lupus erythematosus*	Disturbed glycolysis and mitochondrial oxidative metabolism	Impaired phagocytosis and oxidative burst with increased NETosis and cell death	Altered regulation of mTORC1, AMPK, PPARy	(119, 120)
Rheumatoid arthritis	Altered glucose metabolism	Delayed apoptotic, increased in cytokines spontaneous NETosis	Unregulated PPAR-γ in monocytes is defined, neutrophils?	(121–123)
Atherosclerosis	Metabolic disturbance hyperlipidemia and hypercholesterolemia	Reduced neutrophil recruitment, high susceptibility to inflammation	Reduce neutrophil infiltration by inhibition of NAMPT enzyme	(124, 125)
G6PD deficiency	Compromised PPP metabolism, decrease in NADPH, depletion of glutathione	Defective superoxide generation, NETosis and microbicidal activity	Neutrophil functions depend on severity of G6PD deficiency, regulators?	(40, 55, 126)

*AMPK, AMP-activated protein kinase; ATP, Adenosine triphosphate; FAO, Fatty acid oxidation; G-CSF, Granulocyte-colony stimulating factor; Glut1, glucose transporter; G6P, glucose-6-phosphate; G6Pase-β, glucose-6-phosphatases- β; G6PD, glucose-6-phosphate dehydrogenase; G6PT, glucose-6-phosphate transporter; HIF-1α, Hypoxia-inducible factor-1α; mTOR, Mammalian target of rapamycin; mTORC1, Mammalian target of rapamycin complex 1; NADPH, reduced form of Nicotinamide adenine dinucleotide phosphate; NAMPT, Nicotinamide phosphor-ribosyltransferase; PiT1, Phosphate transporter 1; PPAR-γ, Peroxisome proliferator-activated receptor-gamma; PPP, Pentose phosphate pathway; ROS, Reactive oxygen species*.

### Sepsis

Severe sepsis, a systemic inflammatory syndrome, is associated with impaired neutrophil migration and functions (127–129). Higher expression of chemokines and anti-apoptotic protein Bcl-xL and Mcl-1 during sepsis has been observed to delayed neutrophil apoptosis (127, 128). Defective neutrophil chemotaxis in sepsis has also been associated with reduced expression of chemokine receptor CXCR2, GPCR receptor kinase (GRK), and defective actin assembly (127, 129). Sepsis, due to persisting catabolism, alters cellular bioenergetics and metabolic milieu (111, 112). Role of mitochondrial activity, especially complex V (ATP synthase) and ETC complex III and IV, was associated with LPS-mediated dysfunction of neutrophil chemotaxis (108, 130), which was prevented by AMPK activator and GSK3β inhibitor (108, 130). Inhibition of chemotaxis via upregulation of PPAR-γ under septic conditions was reversed by PPAR-γ inhibition (107).

In yet another study, patients who survived sepsis were presented with increased autophagy that primed NETs formation (131). While dysregulated autophagy and reduced NETosis was evident in sepsis patients who did not survive (131). Consistently, augmentation of autophagy in a mouse model of sepsis also improved survival via a NETs-dependent mechanism (131). Together, functional rejuvenation of neutrophils during sepsis by autophagy provides diverse breakdown products and building blocks to modulate neutrophil metabolism. Importantly, Sumi et al. using a mouse model of cecal ligation and puncture have described the requirement of plasma ATP in neutrophil activation during sepsis (132). Conversely, systemic ATP in sepsis reduced neutrophil activation and chemotaxis by disrupting endogenous purinergic signaling mechanisms (113). Furthermore, suramin, P2-receptor antagonist blocked endogenous ATP signaling and impaired neutrophil activation and chemotaxis leading to increase in bacterial growth and mortality, while removal of systemic ATP by apyrase, ATP-diphosphatase improved bacterial clearance and survival in sepsis, advocating diverse approaches targeting the purinergic signaling for sepsis and other systemic inflammatory disorders (113).

### Diabetes

Diabetes, a metabolic disorder lead to increase the systemic glucose levels, is associated with enhanced susceptibility for infections and defective neutrophil functions. Neutrophil adhesion, chemotaxis, phagocytosis, ROS production, and microbicidal activity defects have been observed in diabetic patients and animal models (114, 115). Hyperglycemia leads to diminished enzymatic activities of glucose-6-phosphate dehydrogenase, and glutaminase, while levels of phosphofructokinase (PFK) are augmented (114). Reduction in G6PD activity adversely affects pentose-phosphate pathway progression and also neutrophil functions (114, 133). Moreover, insulin improves neutrophil phagocytosis and ROS production ability even in the absence of normalized glycemic index, suggesting toward direct effect of insulin on neutrophils (114). Interestingly, under resting state neutrophils express glucose transporters GLUT1 and GLUT3 on surface that remain insensitive to insulin for glucose uptake (25). While in response to phorbol myristate acetate activation, GLUT4 mobilized to the cell surface in an insulin-sensitive manner (25). Defective glucose and glutamine metabolism during hyperglycemia seem to activate a putative compensatory FAO utilization by neutrophils (114). Moreover, enhanced levels of circulating FFA and triacylglycerols cause insulin resistance and also neutrophilic inflammation (53, 54). Neutrophils from streptozotocin-induced diabetic rats exhibit defective autophagy, depolarized mitochondrial potential, and low ATP levels (116). Homocysteine, a sulfur-containing amino acid, levels increased significantly during diabetes and also induced NETosis (134). Furthermore, homocysteine has been observed to enhance the rate of both oxidative phosphorylation and glycolysis along with accumulation of the PPP intermediates in the B cells through pyruvate Kinase M2 (PKM2) upregulation (135). Further investigations are required to understand the metabolic regulations in neutrophils during initiation, development, and progression of diabetes/insulin resistance.

### Cystic Fibrosis (CF)

Cystic fibrosis is a genetic disorder presented with recurrent respiratory tract infections. CF disease is also associated with large numbers of functionally dysregulated neutrophils (117, 136, 137). In CF, neutrophils fail to clear infections, due to functional reprogramming, as well as a reduction in CD16 and CD14 receptors (109). Moreover, increased lipid raft assembly, mobilization of granules and CD11b, CD66 expression were also observed, while elevated mTOR signaling suggested toward anabolic programming (109). Interestingly, CF airway neutrophils adapt to excessive free glucose and amino acids by substantially enhancing the expression of Glut1 and PiT1 (inorganic phosphate transporters) (118). However, it is not clear that how these adaptations affect neutrophil functionality in CF.

### Glucose-6-Phosphate Dehydrogenase (G6PD) Deficiency

G6PD is an essential enzyme that switches glucose metabolism toward PPP (40), by catalyzing the conversion of glucose-6-phosphate to 6-phospho-D-glucono-1,5-lactone and NADPH, that fuels NOX2. G6PD deficiency is a common inherited disorder associated with a decrease in NADPH production leading to the depletion of glutathione, and hemolytic anemia. World Health Organization (WHO) has classified G6PD deficiency into different categories according to the residual G6PD activity and severity of hemolytic anemia (138). Interestingly reduced NOX2 activity strongly correlates with the levels of G6PD activity. G6PD deficiency mimics mild chronic granulomatous disease condition due to defects in the microbicidal and metabolic activity of neutrophils (126). A study using 37 G6PD patients clearly demonstrated a link between G6PD levels and superoxide generation in the neutrophils (139). On the contrary, Ardati et al. did not observe defects in ROS levels as detected by NBT assay in neutrophils having only 23% of normal G6PD activity (140). Conversely, almost 90% loss in G6PD activity led to reduction in ROS generation, NETosis and enhanced susceptibility to infections (55). Consistent to this, mice with hematopoietic G6PD deficiency exhibited improved glucose tolerance, insulin sensitivity, reduction in circulating FFAs, is most likely due to ameliorated inflammation (56). Together, these studies indicate impact of PPP on neutrophil NOX-2 activity and susceptibility to infections in G6PD deficiencies.

### Glycogen Storage Diseases (GSD)

Glycogen is stored in the neutrophil cytosol (7, 141) and glycogenesis (glucose to glycogen conversion) is signaled by the abundance of glucose and ATP. While low glucose conditions, produce glucose-6-phosphate (G6P) through glycogenolysis. G6P is further hydrolyzed to glucose by glucose-6-phosphatases (G6Pase) by gluconeogenesis (58). Glycogen- glucose homeostasis is thus regulated by multi-enzyme glucose-6-phosphatases (G6Pase) complex that includes hydrolase and glucose-6-phosphate transporter (G6PT) subunits. G6PT localizes in the endoplasmic reticulum membrane and translocates G6P from the cytoplasm into the ER lumen, where hydrolase generates glucose. In neutrophils, cycling of G6P/glucose between cytoplasm and ER is utilized for glycolysis and PPP (58). Disturbance in its homeostasis causes GSD, particularly by mutations of G6Pase-α in GSD type Ia (GSD-Ia). GSD-Ia mice as well as patient exhibited elevated number of myeloid progenitor cells, and high G-CSF leading to neutrophilia, however neutrophil function remained unaltered (142). While GSD-Ib is due to the deficiency of G6PT, and it is associated with neutropenia, and neutrophil dysfunction (57, 143). Neutrophils from GSD-Ib patients exhibit defective glucose uptake and reduced levels of G6P, lactate, ATP, and NADPH, even though Glut-1, HIF1-α, and hexokinase (HK-3) expression were augmented (57). Moreover, treatment with PPARγ antagonist, GW9662, rescued neutrophil dysfunction in GSD-Ib patients (57). Similarly, mutations of G6Pase-β (encoded by G6PC3) also cause severe congenital neutropenia syndrome due to enhanced apoptosis (59). Furthermore, G-CSF mitigated neutropenia in G6Pase-β (G6PC3) deficiency by inhibiting apoptosis and inducing glucose uptake and modulating energetics (60). G-CSF also rescued defective superoxide generation, chemotaxis and calcium flux in *G6pc3*^−/−^ neutrophils (60). These studies highlight importance of glycogenolysis mediated metabolic adaptation during neutrophil differentiation and functions through HIF-1α and PPAR-γ. Interestingly, these transcription factors also regulate FAO, TCA, and OXPHOS pathways.

### Systemic Lupus Erythematosus

Systemic lupus erythematosus (SLE) is an autoimmune disease with a high degree of immuno-inflammation and self-destruction. Interestingly, neutrophils in SLE disease condition display dysregulated aggregation, phagocytosis, oxidative burst, and enhanced cell death, NETosis (119). In addition, neutrophils modulate response of other immune cells by releasing self-DNA-peptide complexes that contain immunogenic complexes composed of neutrophil-derived antimicrobial peptides and chromatin (144, 145). Furthermore, SLE is also associated with defective clearance of NETs, however role of NETosis remain to be investigated in inducing autoimmunity (146). Importantly, low-density granulocytes (LDGs), a distinct subset of neutrophils with high levels of pro-inflammatory cytokines were seen elevated in SLE patients (147). Metabolic alteration in T and B cells in SLE has also been described (148). Increase in glycolysis and mitochondrial oxidative metabolism was found in CD4+ T cells in lupus (149). Treatment with 2-DG, metformin or rapamycin rescued lupus symptoms in mice model (149). Ribonucleoprotein immune complexes (RNP-ICs) are commonly found in SLE, which induce NETosis through mitochondrial ROS formation (120). Indeed, RNP-ICs, induced hypopolarization of mitochondria and their translocation to the cell surface. It is important to mention that RNP-ICs also induce NETosis in CGD patients with the consistent development of autoimmunity (120). Metabolic status of neutrophils in SLE has not been explored, even though there is enhanced NETosis, release of self-DNA–peptide complexes and pro-inflammatory phenotype with mitochondrial and mTOR alteration.

### Rheumatoid Arthritis

Another chronic autoimmune disease, rheumatoid arthritis (RA) is presented with extensive activation of the immune system causing synovitis and joint destruction. Neutrophils infiltration in the joint synovial fluid play an essential role in RA particularly during the early stages of the disease by releasing cytotoxic proteases and generating ROS (150). Neutrophils in the synovial fluid also exhibit distinct characteristics like the expression of cytokines and MHC molecules, that are in general expressed on macrophages and dendritic cells and their apoptosis is delayed (121, 122). Furthermore, the survival of SF neutrophils could be due to the hypoxic conditions prevailing in joints afflicted with RA (151). In addition, similar to SLE disease, in RA neutrophils also exhibit increased expression of inferron genes (152) and spontaneous NETosis (123). Furthermore, RA patients with altered glucose metabolism usually have a high risk of diabetes due to insulin resistance and impaired beta cell function (153). Neutrophil metabolic regulations in rheumatoid arthritis also remain less investigated.

### Atherosclerosis

Atherosclerosis is characterized by the plaques accumulated with low-density lipoproteins (LDLs) and cholesterol in the blood vessels in association with hyperlipidemia and inflammation. Researches in the last decade have established presence as well as causative role of neutrophils in atherosclerosis (124, 154). Metabolic disturbance including hyperlipidemia and hypercholesterolemia induce neutrophilia to trigger atherosclerosis (124). Moreover, oxidized-LDL, as well as cholesterol crystals also induce NETosis (82, 155) and inhibition of NAMPT, the enzyme responsible for NAD+ biosynthesis reduces neutrophil infiltration in the atherosclerotic plaque by decreasing CXCL1 levels in ApoE^−/−^ mice (125). Together, metabolic changes in the atherosclerotic environment seem to modulate neutrophil functions, warranting more investigations for establishing the role of neutrophils in initiation/sustenance of atherosclerosis and plaque stability.

## Neutrophil Heterogeneity, Plasticity, and Metabolic Regulation

Recent research has highlighted heterogeneity and plasticity in neutrophil populations on the basis of their state of maturation, altered life span, release of cytokines like IL-10, IL-17A, antigen presentation, diverse surface proteins, distinct antibacterial responses, pro-inflammatory, pro-angiogenic, or immunosuppressive nature (9–12, 147, 156–159). Importantly, microenvironment seems to play a defining role in the neutrophil heterogeneity. For example, tumor-associated neutrophils (TANs) exhibit two distinct phenotypes, N1 cells with anti-neoplastic activity and N2 cells with immunosuppressive characteristics. Transforming growth factor (TGF)-β in the neoplasm microenvironment induces N2 population, which promote tumor development (157). In yet another study, a unique population of neutrophil was identified, expressing transcription factor RORγt and IL-17A after treating with IL-6 and IL-23 (156). Cellular and functional diversity, as well as neutrophil heterogeneity has been majorly found during inflammatory conditions and infectious disorders like sepsis, diabetes, rheumatoid arthritis, and SLE. Interestingly, during infectious and SLE conditions there is an increase in the number of neutrophils expressing programmed death-ligand 1 (PD-L1) (158, 159) and frequency of PD-L1 expressing neutrophils correlates with SLE disease severity (159). These neutrophils also have immunosuppressive characteristics and possibly exert lesser tissue damage. PD-L1^+^ neutrophils on the other hand suppress T-cell immunity and enhance tumor progression (160). A recent study revealed enhanced migration of neutrophils during early-stage of cancer (161) and these neutrophils exhibit high rates of oxidative phosphorylation and glycolysis and produce more ATP (161). Furthermore, tumor microenvironment induces metabolic adaptation in neutrophils with an increase in mitochondrial fitness and immune- suppression (72). Neutrophils in tumor-elicited environment, due to limited glucose supply adapt to mitochondrial FAO to support NOX-2 -dependent ROS production (72). Incidentally, neutrophils depend on glycolysis during early phase of intense ROS generation, while during late phase ROS production is supported by mitochondrial FAO (72). Taken together, recent developments unravel metabolic decisions in cellular plasticity and differential pro-inflammatory or immunosuppressive responses. Future studies to provide better understanding of neutrophil heterogeneity and their metabolic adaptations to perform microenvironment specific function are now needed.

## Concluding Remarks and Perspectives

Neutrophils pave the way for immunoregulation at the site of infection/inflammation. Recent research in the area of neutrophil biology has challenged the hypothesis of neutrophils dependence on glycolysis for their varied functional responses, differentiation, and survival. Neutrophils however utilize diverse metabolic pathways including glycolysis, glutaminolysis, PPP, FAO, TCA cycle to perform distinct functions. Thus, targeting these metabolic adaptations by neutrophils might be beneficial for combating infections and inflammation mediated tissue damage. Furthermore, metabolic switch(es), if any, remains least defined so far to impact neutrophil functionality and heterogeneity. Cutting-edge single cell metabolomics analyses in coordination with high-throughput approaches might be of immense importance to investigate heterogeneity in neutrophil population (162). Detailed untargeted metabolomics studies might help in identifying metabolic switch(es) during the apoptotic program and/or enhanced survival. It would also be interesting to decipher how neutrophil adapt to metabolism during various pathological conditions and hypoxia due to availability of distinct nutrients, metabolic intermediates, and a mixture of various mediators (see Outstanding Questions). Finally, improved understanding of the metabolic switch(es) in neutrophil populations migrating to inflammatory/infectious sites and survive at these sites could be helpful in unraveling the mechanistic understanding of various disorders.

## Outstanding Questions

- Is there any specific role of metabolic adaptation(s) if any during pro-inflammatory and immunosuppressive conditions in the various neutrophil subsets?
- Can distinct metabolic pathways be efficiently targeted/suppressed to modulate specific neutrophil functions and survival?
- Can neutrophils subsets be targeted to tackle infectious and inflammatory conditions?
- Can single cell metabolomics be helpful in understanding and defining the neutrophil plasticity?
- Metabolic activities in the subcellular organelle and phagosomes of the resting and activated neutrophils remain undefined so far.
- Comparison of metabolic pathways in neutrophils with other phagocytic cells is of fundamental importance to delineate the role of metabolism if any in the short-lived fate of neutrophils.

## Author Contributions

SK conceptualized the idea, collects relevant information, prepared figures, table, wrote the manuscript. MD provided critical suggestion and helped in writing the manuscript.

### Conflict of Interest Statement

The authors declare that the research was conducted in the absence of any commercial or financial relationships that could be construed as a potential conflict of interest.
